# The Association of HLA-B*35 and *GSTT1* Genotypes and Hepatotoxicity in Thai People Living with HIV

**DOI:** 10.3390/jpm12060940

**Published:** 2022-06-08

**Authors:** Noppadol Chanhom, Jiraphun Jittikoon, Sukanya Wattanapokayakit, Surakameth Mahasirimongkol, Angkana Charoenyingwattana, Wanvisa Udomsinprasert, Usa Chaikledkaew, Supharat Suvichapanich, Taisei Mushiroda, Sasisopin Kiertiburanakul, Archawin Rojanawiwat, Wittaya Wangsomboonsiri, Weerawat Manosuthi, Pacharee Kantipong, Anucha Apisarnthanarak, Wilawan Sangsirinakakul, Pawinee Wongprasit, Romanee Chaiwarith, Woraphot Tantisiriwat, Somnuek Sungkanuparph, Wasun Chantratita

**Affiliations:** 1 Department of Biochemistry, Faculty of Pharmacy, Mahidol University, Bangkok 10400, Thailand; noppadol.cha@mahidol.ac.th (N.C.); wanvisa.udo@mahidol.ac.th (W.U.); supharat.suv@mahidol.ac.th (S.S.); 2 Genomic Medicine Centre, Division of Genomic Medicine and Innovation Support, Department of Medical Sciences, Ministry of Public Health, Nonthaburi 11000, Thailand; sukanya.w@dmsc.mail.go.th (S.W.); surakameth.m@dmsc.mail.go.th (S.M.); 3 Genomic Medicine Center, Faculty of Medicine Ramathibodi Hospital, Mahidol University, Bangkok 10400, Thailand; angkana.chr@gmail.com (A.C.); wasun.cha@mahidol.ac.th (W.C.); 4 Social Administrative Pharmacy Division, Department of Pharmacy, Faculty of Pharmacy, Mahidol University, Bangkok 10400, Thailand; usa.chi@mahidol.ac.th; 5 Laboratory for Pharmacogenomics, RIKEN Center for Integrative Medical Sciences, Yokohama 230-0045, Japan; mushiroda@riken.jp; 6 Department of Medicine, Faculty of Medicine Ramathibodi Hospital, Mahidol University, Bangkok 10400, Thailand; sasisopin.kie@mahidol.ac.th; 7 Clinical Research Center, Department of Medical Sciences, Ministry of Public Health, Nonthaburi 11000, Thailand; archawin.r@dmsc.mail.go.th; 8 Department of Internal Medicine, Sawanpracharak Hospital, Nakornsawan 60000, Thailand; mdwittaya@yahoo.com; 9 Internal Medicine, Bamrasnaradura Infectious Diseases Institute, Nonthaburi 11000, Thailand; idweerawat@yahoo.com; 10 Department of Internal Medicine, Chiangrai Prachanukroh Hospital, Chiang Rai 57000, Thailand; pachareek@hotmail.com; 11 Division of Infectious Diseases, Department of Medicine, Thammasat University Hospital, Pathum Thani 12121, Thailand; anapisarn@yahoo.com; 12 Internal Medicine, Maharaj Nakorn Ratchasima Hospital, Nakorn Ratchasima 30000, Thailand; noonmed@yahoo.com; 13 Internal Medicine, Buriram Hospital, Buri Ram 31000, Thailand; pawiwong@yahoo.com; 14 Department of Medicine, Faculty of Medicine, Chiang Mai University, Chiang Mai 50200, Thailand; romanee.c@cmu.ac.th; 15 Department of Preventive Medicine and Social Medicine, Faculty of Medicine, Srinakharinwirot University, Bangkok 10110, Thailand; woraphot@pol.net; 16 Chakri Naruebodindra Medical Institute, Faculty of Medicine Ramathibodi Hospital, Mahidol University, Samut Prakan 10540, Thailand; somnuek.sun@mahidol.ac.th

**Keywords:** drug-induced liver injury, glutathione s-transferase, human immunodeficiency virus, hepatotoxicity, adverse drug reaction, genetic polymorphisms

## Abstract

Glutathione s-transferase (GST) is a family of drug-metabolizing enzymes responsible for metabolizing and detoxifying drugs and xenobiotic substances. Therefore, deletion polymorphisms of *GST*s can be implicated in developing several pathological conditions, including antiretroviral drug-induced liver injury (ARVDILI). Notably, *GST* polymorphisms have been shown to be associated with ARVDILI risk. However, data on *GST* polymorphisms in the Thai population are limited. Therefore, this study investigated possible associations between *GST* genetic polymorphisms and ARVDILI development. A total of 362 people living with HIV (PLHIV) and 85 healthy controls from multiple centers were enrolled. *GSTM1* and *GSTT1* genetic polymorphisms were determined using polymerase chain reactions. In addition, HLA genotypes were determined using a sequence-based HLA typing method. After comparing *GST* genotypic frequencies, there was no significant difference between PLHIV and healthy volunteers. However, while observing the PLHIV group, *GSTT1* wild type was significantly associated with a 2.04-fold increased risk of ARVDILI (95%CI: 1.01, 4.14; *p* = 0.045). Interestingly, a combination of *GSTT1* wild type and HLA-B*35:05 was associated with a 2.28-fold higher risk of ARVDILI (95%CI: 1.15, 4.50; *p* = 0.02). Collectively, *GSTT1* wild type and a combination of *GSTT1* wild type plus HLA-B*35:05 were associated with susceptibility to ARVDILI in the Thai population.

## 1. Introduction

Since the discovery of the human immunodeficiency virus (HIV) in 1983 [1], the virus has spread across the globe, becoming one of the most life-threatening diseases. Increased incidences of HIV-related hospitalizations and deaths commonly occur due to prolonged immune deficiency associated with a decrease in CD4^+^ lymphocytes, leading to other disease complications. Although receiving antiretroviral (ARV) treatment can help mitigate the disease severity, patients can develop adverse drug reactions, such as hepatotoxicity, commonly known as ARVDILI [2]. Unfortunately, Reister et al. reported that 8.9–10.8% of patients have nevirapine (NVP) hepatotoxicity [3]. Interestingly, genetic polymorphism has been reported as one of the possible factors associated with the development and progression of HIV infection and ARVDILI [4,5], thereby highlighting the possibility of genetic variations as alternative markers for ARVDILI in PLHIV.

GSTs are phase II metabolizing enzymes for drug detoxification through conjugation specific to glutathione (GSH). They are gaining increasing interest as a cellular defender against drugs, carcinogens, and oxidative stress caused by excessive reactive oxygen species (ROS) production. *GSTM1* and *GSTT1* are particularly fascinating *GST* genes because these two genes were defined as polymorphic in humans [6]. Given that *GSTM1* is located on chromosome 1p13.3, it has been shown that deletion of this gene can impair an individual’s ability to detoxify some carcinogens, ROS, or certain drugs [7,8]. The *GSTT1* gene is located on chromosome 22q11.23. This enzyme participates in detoxifying drugs and their conjugation with electrophilic and hydrophobic compounds [9,10]. In PLHIV, it has been recognized that GSTs are responsible for mitigating cellular damage resulting from oxidative stress via conjugating glutathione to ROS [11]. Accordingly, investigating the influence of *GST*s’ genetic polymorphisms on ARV-administered patients may provide insight into the safe use of this medication in HIV-infected individuals.

In addition to *GST* polymorphisms, human leukocyte antigen (HLA) has been reportedly associated with NVP rash and/or hepatitis across different populations [12,13,14,15,16]. For example, in the Thai population, Chantarangsu et al. demonstrated a strong association between HLA-B*35:05 and NVP-induced skin rash [13]. Moreover, a recent study illustrated severe cutaneous adverse reactions were frequently comorbid with hepatitis, especially acute generalized exanthematous pustulosis and drug reaction with eosinophilia and systemic symptoms [17]. It has been hypothesized that there is a significant association between specific HLA genotypes and hepatotoxicity in Thais.

Although a correlation between *GST* gene deletion and the risk of developing ARVDILI has been explored in the Indian population [5], to the best of our knowledge, no published studies have yet examined associations between *GST* and *HLA* polymorphisms with the risk of developing ARVDILI in the Thai population. Accordingly, the objective of this study was to determine whether genetic polymorphisms of *GSTs* or *HLA* were associated with ARVDILI risk in Thai PLHIV.

## 2. Materials and Methods

### 2.1. Study Subjects

This observational multicentered retrospective case-control study consisted of two study subject groups. The first group included newly diagnosed Thai HIV-positive patients being treated with NVP-based HIV treatment at Ramathibodi Hospital (Bangkok, Thailand), Bamrasnaradura Infectious Diseases Institute (Nonthaburi, Thailand), HRH Princess Maha Chakri Sirindhorn Medical Center (Nakhon-Nayok, Thailand), Thammasat University Hospital (Pathum-Thani, Thailand), Chiangrai Prachanukroh Hospital (Chiangrai, Thailand), Maharat Nakhon Ratchasima Hospital (Nakhon-Ratchasima, Thailand), Sawanpracharak Hospital (Nakhonsawan, Thailand), Buriram Hospital (Buri-Ram, Thailand), or Maharaj Nakorn Chiang Mai Hospital (Chiang-Mai, Thailand). The other was a healthy control group recruited from Ramathibodi Hospital (Bangkok, Thailand) from 2010 to 2012. This study protocol was performed in compliance with the International Guidelines for Human Research Protection, including the Declaration of Helsinki, and the Belmont Report. It was approved by the Institutional Review Board of the Faculty of Dentistry/Faculty of Pharmacy, Mahidol University (IRB No. 2020/PY067). Written informed consent was obtained from all patients before their admission to the study.

**Figure 1:**
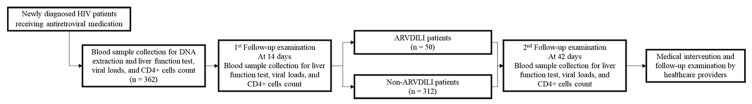
Timeline for patients’ recruiting process. ARVDILI, antiretroviral drug-induced liver injury; HIV, human immunodeficiency virus.

Four hundred and forty-seven study participants, including 362 PLHIV and 85 healthy controls, were enrolled (Figure 1). PLHIV were categorized into 50 ARVDILI cases and 312 non-ARVDILI cases. The inclusion criteria for non-ARVDILI PLHIV were as follows: (i) the patient agreed to sign an informed consent form after thoroughly considering the study protocol, (ii) male or female patients aged between 18–70 years old, (iii) a certified laboratory procedure confirmed the patient with HIV-1 infection, and (iv) the patient has never been received any antiretroviral treatment medication. The exclusion criteria for this group were as follows: (i) the patient requested to be excluded from the study, (ii) HIV-infected patient must breastfeed, (iii) the patient has ever received other HIV treatment medication, (iv) the patient’s liver enzymes were more than five times the upper limit of normal. Under Thailand National Guidelines on HIV/AIDs Diagnosis, Treatment, and Prevention 2010 [18], the inclusion criteria for hepatotoxicity were as follows: (i) the HIV patients who presented with hepatotoxicity symptoms, such as nausea, vomiting, fatigue, and myopathy, and had aminotransferase (AST) or alanine aminotransferase (ALT) levels higher than five times the upper limit of normal (ULN); (ii) HIV patients who had AST or ALT level of 5–10 times the ULN without presenting with symptoms. The exclusion criteria for the hepatotoxicity group were as follows: (i) the patient had concomitant administration of other potentially hepatotoxic drugs, as defined by the LiverTox database [19]; (ii) the patient had an underlying disease such as viral hepatitis, liver cirrhosis, hepatoma, or tuberculosis infection. The inclusion criteria for healthy control were as follows: (i) the volunteer agreed to sign an informed consent form after thoroughly considering the study protocol; (ii) male or female individuals aged 18–70 years old; (iii) the individual had never been infected with HIV-1. The exclusion criteria for this group were as follows: (i) the patient had requested to be excluded from the study. All PLHIV were treated under the Thailand National Guidelines on HIV/AIDs Diagnosis, Treatment, and Prevention 2010 [18]. In addition, clinical data and blood samples of the patients were collected by onsite associates and recorded in the project’s specific pre-defined clinical record forms.

### 2.2. DNA Sample Retrieval and DNA Quantification

As previously described, the leftover DNA samples were retrieved from Ramathibodi hospital [13]. Extracted DNA was analyzed using agarose gel electrophoresis and quantitatively quantified by ultraviolet spectrometer Nanodrop^®^ 2000c (Thermo Scientific, Waltham, MA, USA). Furthermore, each hospital’s automated machine routinely measured all clinical parameters including CD4+, viral load, liver function test, pregnancy test, and HIV drug resistance.

### 2.3. Genetic Genotyping and Data Retrievals

*GSTM1* and *GSTT1* genotypes were determined using PCR. β-globin was used as an internal standard. The primers used for *GSTM1* and *GSTT1* were as follows: *GSTM1* forward 5′–GAACTCCCTGAAAAGCTAAAGC–3′; *GSTM1* reverse 5′–GTTGGGCTCAAATATACGGTGG–3′; *GSTT1* forward 5′–TTCCTTACTGGTCCTCACATCTC–3′; *GSTT1* reverse 5′–TCACCGGATCATGGCCAGCA–3′ [20]. The PCR was performed with 20 ng of genomic DNA in a total volume of 20 μL using a T100 Thermal Cycler (BIORAD, Hercules, CA USA) and KAPA2G Fast Multiplex PCR Kit (KAPA Biosystems, Wilmington, MA, USA), with an initial denaturation step of 95 °C for 5 min, followed by 35 cycles of denaturation at 95 °C for 15 s, annealing at 63 °C for 20 s, and extension at 72 °C for 20 s. The PCR products were analyzed on 2% agarose gel pre-stained with ethidium bromide. *GSTM1* polymorphism was identified by the presence or absence of bands at 219 base pairs, whereas *GSTT1* polymorphism was interpreted by the presence or absence of bands at 459 base pairs (Figure S1). Moreover, both *GSTM1* and *GSTT1* genotypic data from healthy controls were derived from whole-genome sequencing data using ClinCNV software [21]. *HLA* genotypic data from a previous study were retrieved for analytical purposes [13].

### 2.4. Data Analysis

Statistical analyses were executed by Statistical Package for Social Sciences version 22.0 (SPSS, Inc., Chicago, IL, USA). Quantitative parameters, such as demographics and clinical data, normally distributed, were compared between groups using χ^2^ tests and Student’s *t*-test, where appropriate. Comparisons in non-normally distributed continuous data among each group were evaluated by the Mann–Whitney U test or Kruskal–Wallis H test. Statistical differences in genotypic distributions of cases and controls were undertaken using Pearson’s Chi-square or Fisher’s exact test. The Mantel–Haenszel statistic method was used to combine the effects of genetic polymorphisms.

## 3. Results

### 3.1. Demographic and Clinical Characteristics of PLHIV

As detailed in Table 1, the demographic data before ARV treatment, 14 days after ARV treatment, and 42 days after ARV treatment were compared between ARVDILI or HIV+ with hepatotoxicity and non-ARVDILI or HIV+ without hepatotoxicity groups. There were no differences in demographic data, including age, sex, BMI, smoking, alcohol consumption, and received drug regimen between PLHIV with and without ARVDILI. In addition, we observed the viral loads and CD4+ T-cells count both before ARV treatment and 42 days after ARV treatment. The results indicate no difference between the ARVDILI and non-ARVDILI groups. Clinical parameters, including AST and ALT, were significantly lower in PLHIV without ARVDILI than in patients with ARVDILI (*p* < 0.001).

### 3.2. Genotypic Distributions of GSTs and HLA-B among Healthy Volunteers, ARVDILI and Non-ARVDILI Groups

When frequencies of *GSTM1* and *GSTT1* polymorphisms between healthy control and PLHIV were compared, no significant differences in *GSTM1* and *GSTT1* polymorphisms between groups were observed (Table 2). Although there were significant differences in levels of liver function tests between ARVDILI and non-ARVDILI groups after 14 and 42 days of HIV treatment, as demonstrated in Table 3, we found that *GSTT1* wild type was significantly more prevalent in the ARVDILI group, with an odds ratio (OR) of 2.04 (95% CI, 1.01–4.14; *p* = 0.045) (Table 3). In addition to *GST* distributions, HLA-B*35:01, HLA-B*35:03, HLA-B*35:05, HLA-B*35:60, and HLA-B*58:01 were not significantly associated with ARVDILI, even though HLA-B*35:01 was predominant in the ARVDILI group.

### 3.3. Effects of Multiple Genes on ARVDILI

**Figure 2:**
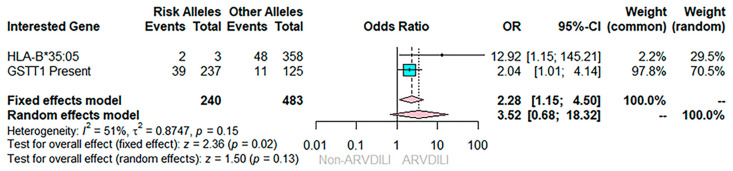
Effects of *GSTT1* and HLA-B*35:05 gene polymorphisms on ARVDILI combination using a fixed-effect model of Mantel–Haenszel statistics. The cyan squares and lines represents the point estimate of the odd ratio and 95% confidence intervals, while the pink diamonds represents the overall effect of all studies using the fixed and random effect models, respectively.

In this study, we aimed to observe the effect of multiple genes. Therefore, Mantel–Haenszel statistical analysis was applied in order to investigate the additional effect of interesting genes. After pooling the effects of *GSTT1* and HLA-B*35:05 using fixed-effect Mantel–Haenszel statistics, results revealed that a combination of *GSTT1* wild type and HLA-B*35:05 was significantly associated with a 2.28-fold increased risk of ARVDILI in the Thai population (pooled OR = 2.28; 95% CI: 1.01–4.14; *p* = 0.02), as illustrated in Figure 2.

## 4. Discussion

HIV is widely recognized as one of the most affecting and life-threatening infectious diseases, impacting millions of individuals globally and hundreds of thousands in Thailand [22]. PLHIVs die and are hospitalized for various causes, including a weakened immune system, which eventually results in an increased risk of contracting opportunistic infections, malignancy, and even suicide [23]. Therefore, ARV therapy is an efficient strategy to mitigate HIV-related mortality to reduce the disease severity and increase survival chances. However, another cause of mortality in PLHIV is adverse drug reactions caused by ARV therapy or HIV treatment, such as ARVDILI [24,25]. In that context, the novel strategy for predicting ARVIDILI risk is critical for long-term ARV prescription in various populations. Unfortunately, research on genetic variations associated with ARVDILI is sparse. They may, however, potentially serve as genetic biomarkers for identifying not only severe patients, but also ARVDILI development in PLHIV. GSTs and HLA, which are phase II xenobiotic-metabolizing enzymes and immune regulators, respectively, are exciting candidates for investigating associations between their polymorphisms and ARVDILI.

The present study is the first to uncover that the frequencies of *GSTM1* and *GSTT1* null genotypes in the Thai healthy population were 52.9% and 32.9%, respectively. Consistent with our results, Kasthurinaidu et al. revealed that *GSTM1* deletion frequency was not equally distributed by race [26]. Besides this, another study by Lam et al. demonstrated that *GSTT1* null frequency was not equally distributed by race [27]. In line with our results, we found no significant differences in frequencies of *GST*s deletions between healthy controls and PLHIV. This result led us to speculate that Thai people may be more prone to some adverse reactions due to medications, cancers, and oxidative stress [7,8,28] as a result of the high prevalence of *GSTM1* null alleles in the Thai population.

Another striking aspect of *GST*s’ relevance in HIV infection is ARVDILI. According to a previous study by Singh et al., it has been suggested that homozygous deletions of *GSTM1* and *GSTT1* may predict the acquisition of hepatotoxicity in PLHIV receiving ARV [5]. To address this hypothesis, our study investigated this matter and found no correlation between *GST* gene deletions and the risk of developing ARVDILI in Thai PLHIV. The possible reasons for the discrepancy between our findings and Singh et al. might be due to various factors. Firstly, our study included only NVP-based ART patients, while Singh’s study included both nevirapine-based and efavirenz-based ART patients. In addition to the standard regimen, genotypic frequencies of *GST* deletions in the Indian population were lower than those in the Thai population [26], which may contribute to our inconsequential findings.

On the contrary, we discovered that *GSTT1* presence was significantly associated with an increased risk of ARVDILI in the Thai population. As previously mentioned, cells can employ GSTs to conjugate GSH in response to xenobiotics, oxidative stress, or carcinogens. According to Ivanov et al., it has been postulated that prolonged HIV infection may result in massive production of oxidative stress [11]. This mechanism may account for the depletion of GSH in *GSTT1* carriers, which are capable of effectively using GSH in response to oxidative stress and exacerbating hepatocellular damage caused by NVP and its metabolites [29]. In addition to the *GSTT1*-ARVDILI relationship, combining the effects of *GSTT1* wild type and HLA-B*35:05 can yield a greater significant level. This result confirmed the findings of Popovic et al., who demonstrated a correlation between immune response and hepatotoxicity due to NVP [30]. Furthermore, according to Srivastava et al., enhanced hyphenation of tissue proteins by reactive NVP metabolites under a GSH-depleted environment might also lead to the initiation of an immune response [31]. Therefore, treating ARVDILI patients with *N*-acetylcysteine, a precursor to GSH, may help to decrease the severity of this adverse event [32]. Unquestionably, further studies with a larger sample size are necessary to investigate this matter or combine the impacts of genetic polymorphisms in order to clarify our results.

However, it should be noted that this study had some limitations. Firstly, this was a retrospective case-control study that might preclude the determination of cause-and-effect relationships. Therefore, it is recommended that multi-center prospective cohorts are needed to verify any associations. Furthermore, this study could not identify the specific drug-induced liver injury because antiretroviral treatment guidelines indicate all three antiretroviral drugs, two nucleoside reverse transcriptase inhibitors and one non-nucleoside reverse transcriptase inhibitor, can be concurrently delivered to HIV patients, and rechallenge histories are often lacking [18]. On the other hand, the strength of our study is that our findings might be used to support the view that genetic polymorphisms could be used as alternative markers along with a conventional tool for monitoring ARVDILI progression in NVP-administered patients.

## 5. Conclusions

Our study revealed that GSTT1 and HLA-B*35:05 genotypes are associated with ARVDILI in Thai PLHIV, which may help predict ARVDILI risk in the patients and have predictive value as a genetic biomarker. However, further studies are necessary and encouraged to draw a more decisive conclusion and justify using *GSTT1* and HLA-B*35:05 genotypes in combination as an additional genetic biomarker.

## Figures and Tables

**Table 1:** Clinical characteristics of HIV patients with and without ARVDILI.

Variables	Before ART Treatment		*p*-Value	At 14 Days after Treatment		*p*-Value	At 42 Days after Treatment		*p*-Value
	HIV+ without Hepatotoxicity	HIV+ with Hepatotoxicity		HIV+ without Hepatotoxicity	HIV+ with Hepatotoxicity		HIV+ without Hepatotoxicity	HIV+ with Hepatotoxicity	
N (%)	312 (100.0)	50 (100.0)							
Age (years)	36.32 ± 10.29	39.26 ± 11.29	0.080						
Sex (M/F)	180/132	25/25	0.357						
BMI (kg/m^2^)	22.58 ± 21.52	20.52 ± 3.58	0.098						
Smoking (%)	70 (23.0)	9 (18.4)	0.581						
Alcohol (%)	98 (34.4)	16 (35.6)	0.868						
CD4+ (cells/mm^3^)	144.23 ± 105.11	167.49 ± 114.14	0.224	N/A	N/A	N/A	269.12 ± 151.24	303.97 ± 144.38	0.129
Viral load (copies/mm^3^)	129,027.37 ± 261,163.86	105,268.46 ± 170,374.59	0.332	N/A	N/A	N/A	16,760.24 ± 163,799.31	138.63 ± 445.04	0.386
AST (IU/L)	31.74 ± 15.47	32.76 ± 13.42	0.299	28.09 ± 13.58	61.72 ± 78.32	<0.001	28.22 ± 14.84	69.02 ± 108.36	<0.001
ALT (IU/L)	33.48 ± 18.86	36.66 ± 18.33	0.235	32.82 ± 17.67	74.80 ± 102.83	<0.001	35.75 ± 29.40	89.19 ± 144.50	<0.001

Abbreviation: ALT, alanine aminotransferase; ART, antiretroviral therapy; AST, aspartate aminotransferase; BMI, body mass index; HIV, human immunodeficiency virus.

**Table 2:** Genotypic distributions of *GST*s in healthy volunteers and PLHIV.

Genotypic Distribution of *GST*s in Non-ARVDILI and ARVDILI Patients				
*GST*	HIV (*n* = 362)	Healthy (*n* = 85)	OR (95% CI)	*p*-Value
*GSTM1* present	151 (41.7%)	40 (47.1%)	1	ref.
*GSTM1* null	211 (58.3%)	45 (52.9%)	1.257 (0.781–2.022)	0.346
*GSTT1* present	237 (65.5%)	57 (67.1%)	1	ref.
*GSTT1* null	125 (34.5%)	28 (32.9%)	1.097 (0.662–1.817)	0.720
*GSTM1* or *GSTT1* present	292 (80.7%)	71 (83.5%)	1	ref.
*GSTM1* and *GSTT1* null	70 (19.3%)	14 (16.5%)	1.268 (0.668–2.406)	0.467

Abbreviation: ARVDILI, antiretroviral drug-induced liver injury, *GSTM1*, glutathione S-transferase Mu 1; *GSTT1*, glutathione S-transferase theta 1; HIV, human immunodeficiency virus.

**Table 3:** Genotypic distributions of *GST*s and *HLA* in PLHIV with and without ARVDILI.

*GST*	HIV+ with Hepatotoxicity	HIV+ without Hepatotoxicity	Odd Ratio (95% CI)	*p*-Value
N (%)	50 (100%)	312 (100%)		
*GST*				
*GSTM1* present	21 (42.0%)	130 (41.7%)	1	Ref.
*GSTM1* null	29 (58.0%)	182 (58.3%)	0.99 (0.54–1.81)	0.965
*GSTT1* present	39 (78.0%)	198 (63.5%)	1	Ref.
*GSTT1* null	11 (22.0%)	114 (36.5%)	0.49 (0.24–0.99)	0.045
*GSTM1* or *GSTT1* present	44 (88.0%)	248 (79.5%)	1	Ref.
*GSTM1* and *GSTT1* null	6 (12.0%)	64 (20.5%)	0.53 (0.22–1.29)	0.157
*HLA-B*				
*35:01	0 (0.0)	6 (1.9)	0.47 (0.03–8.39)	1.000
*35:03	0 (0.0)	4 (1.3)	0.68 (0.04–12.80)	1.000
*35:05	2 (4.0)	1 (0.3)	12.92 (1.15–145.21)	0.052
*35:60	0 (0.0)	1 (0.3)	2.06 (0.08–51.17)	1.000
*58:01	5 (10.0)	45 (90.0)	1.415 (0.59–3.40)	0.427

Abbreviation: ARVDILI, antiretroviral drug-induced liver injury; *GSTM1*, glutathione S-transferase Mu 1; *GSTT1*, glutathione S-transferase theta 1; HIV, human immunodeficiency virus; Ref, reference.

## Data Availability

The data analyzed and generated during the current study are available from the corresponding author on reasonable request.

## Supplementary Materials

The following supporting information can be downloaded at: https://www.mdpi.com/article/10.3390/jpm12060940/s1, Figure S1. Agarose gel electrophoresis (2% agarose) of PCR amplified products using species-specific PCR primer sets. Lanes 1–24 are examining the presence of GSTM1 and GSTT1 using β-globin as internal control. Lane M, 100 base-pair DNA size marker.
