# Supplementary material for: The Association of HLA-B*35 and GSTT1 Genotypes and Hepatotoxicity in Thai People Living with HIV
Source: J Pers Med. 2022 Jun 8;12(6):940. doi: 10.3390/jpm12060940 (PMC9225434; doi:10.3390/jpm12060940)
Supplement: Supplementary file 1 [file jpm-12-00940-s001.zip › jpm-1731278-supplementary.pdf]

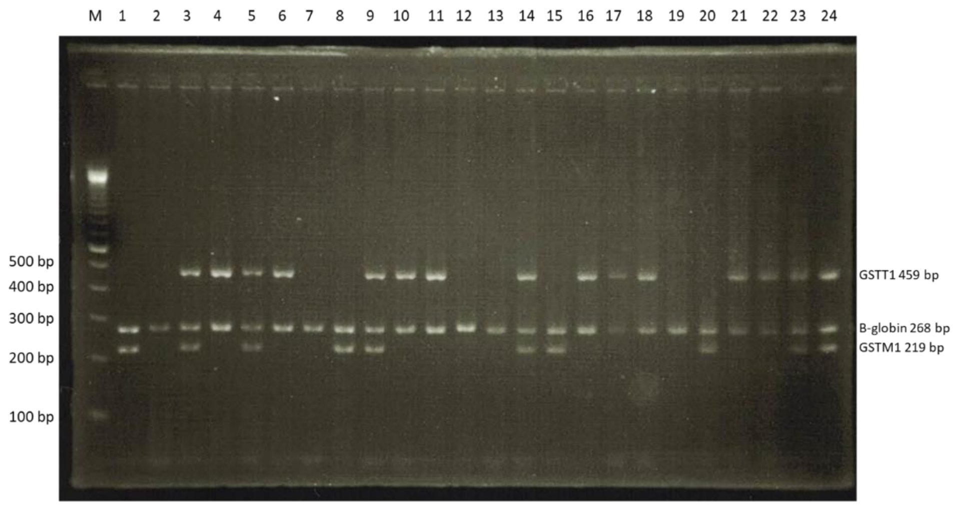

Figure S1. Agarose gel electrophoresis (2% agarose) of PCR amplified products using species-specific PCR primer sets. Lanes 1–24 are examining the presence of GSTM1 and GSTT1 using β-globin as internal control. Lane M, 100 base-pair DNA size marker.
